# Complete Genome Sequence of Bartonella bacilliformis Strain KC584 (ATCC 35686)

**DOI:** 10.1128/MRA.01377-19

**Published:** 2020-01-02

**Authors:** Alexander A. Dichter, Tilman G. Schultze, Sabrina A. Becker, Pablo Tsukayama, Volkhard A. J. Kempf

**Affiliations:** a Institute for Medical Microbiology and Infection Control, University Hospital, Goethe University Frankfurt am Main, Frankfurt, Germany; b Facultad de Ciencias y Filosofía/Instituto de Medicina Tropical “Alexander von Humboldt”, Universidad Peruana Cayetano Heredia, Lima, Peru; Georgia Institute of Technology

## Abstract

Bartonella bacilliformis is the biological agent of Carrion’s disease, a vector-borne, life-threatening human bartonellosis restricted to South America. Here, we report the complete genome sequence of *B. bacilliformis* KC584 (ATCC 35686). Although it is commonly used as a reference strain, to date, its complete genome has not been published.

## ANNOUNCEMENT

Bartonella bacilliformis is a Gram-negative alphaproteobacterium and the causative agent of Carrion’s disease, a vector-borne biphasic illness (1, 2). The pathogen is transmitted by the bite of infected *Lutzomyia* sand flies, which are indigenous to the South American Andes (1, 3). To date, humans are the only known reservoir for *B. bacilliformis* (4). Infections result in two clinical manifestations; in the acute phase, known as Oroya fever, the bacterium infect erythrocytes, causing a severe hemolytic anemia with high fatality rates in untreated patients. The chronic phase, “verruga peruana,” is characterized by the formation of blood-filled hemangioma-like lesions at skin sites caused by bacterially induced abnormal endothelial cell proliferation (1, 2, 5). Draft genomes from clinical isolates have been published using short-read sequencing technologies. However, an assembly of a closed circular contig failed due to read-length limitations (6, 7).

*B. bacilliformis* strain KC584 was originally isolated in 1959 and characterized in greater detail in 1991 (8, 9). Whole-genome sequencing (WGS) was pursued in a hybrid approach of Illumina short reads and PacBio long reads to improve contig assembly. For short-read WGS, *B. bacilliformis* strain KC584 was streaked out from cryostock on Columbia blood agar (Becton, Dickinson, Heidelberg, Germany) and incubated for 4 days at 28°C. Bacteria were collected with swabs, suspended in phosphate-buffered saline (pH 7.0 to 7.3), and pelleted at 10,000 × *g* for 3 minutes. The QIAamp DNA minikit (Qiagen, Hilden, Germany) was used for genomic DNA isolation. A short-read sequencing library was generated utilizing a NEBNext Ultra II FS DNA library prep kit (NEB, Ipswich, MA, USA). Sequencing was carried out on a MiSeq sequencer using v2 chemistry. The paired-end sequencing run (2 × 250 bp) yielded a total of 1,266,387 read pairs.

For long-read WGS, bacteria were cultivated in *Bartonella* liquid medium (10) for 4 days at 28°C and 120 rpm. High-molecular-weight (HMW) DNA was isolated with the Qiagen MagAttract HMW DNA kit and fragmented to 10- to 12-kb fragments using Covaris g-TUBEs (Covaris, Brighton, UK). The sequencing library was prepared using the Pacific Biosciences protocol for preparing multiplexed microbial SMRTbell libraries, barcoded hairpin adapters (IDT, Leuven, Belgium), and a PacBio barcoded adapter. The library was sequenced on a Pacific Biosciences Sequel instrument using v3.0 chemistry, including Sequel Polymerase v3.0 and single-molecule real-time (SMRT) cells v3.

Using circular-consensus sequencing, a total of 2,894,449,675 bases were generated, representing 165,311,302 consensus-corrected bases in 37,022 reads. The read lengths ranged from 600 to 21,000 bp (average, 4,645 bp). Quality control for all sequencing files was performed using FastQC v0.11.8 (11).

For hybrid *de novo* assembly, Unicycler v0.4.8 (12) was run with the default settings, providing a single contig with a circular sequence of 1,411,580 bp and a GC content of 38.2%. As a final assembly control, long reads were mapped against the assembled genome with Minimap2 (13). Annotation was done with the NCBI Prokaryotic Genome Annotation Pipeline (14). Default parameters were used for all software unless otherwise noted.

A publicly available genome sequence for this strain is crucial for understanding its fundamental traits and facilitates further work on a genomic level.

### Data availability.

The closed genome sequence has been submitted to GenBank (accession number CP045671). The associated BioProject and BioSample accession numbers are PRJNA579486 and SAMN13112075, respectively.

## References

[1] Minnick MF, Anderson BE, Lima A, Battisti JM, Lawyer PG, Birtles RJ 2014 Oroya fever and verruga peruana: bartonelloses unique to South America. PLoS Negl Trop Dis 8:e2919. doi:10.1371/journal.pntd.0002919. 25032975 PMC4102455

[2] Maguina C, Garcia PJ, Gotuzzo E, Cordero L, Spach DH 2001 Bartonellosis (Carrión’s disease) in the modern era. Clin Infect Dis 33:772–779. doi:10.1086/322614. 11512081

[3] Schultz MG 1968 A history of bartonellosis (Carrión’s disease). Am J Trop Med Hyg 17:503–515. doi:10.4269/ajtmh.1968.17.503. 4876803

[4] Gomes C, Pons MJ, del Valle Mendoza J, Ruiz J 2016 Carrion’s disease: an eradicable illness? Infect Dis Poverty 5:105. doi:10.1186/s40249-016-0197-7. 27903286 PMC5131403

[5] Sanchez Clemente N, Ugarte-Gil CA, Solórzano N, Maguiña C, Pachas P, Blazes D, Bailey R, Mabey D, Moore D 2012 *Bartonella bacilliformis:* a systematic review of the literature to guide the research agenda for elimination. PLoS Negl Trop Dis 6:e1819. doi:10.1371/journal.pntd.0001819. 23145188 PMC3493376

[6] Guillen Y, Casadellà M, García-de-la-Guarda R, Espinoza-Culupú A, Paredes R, Ruiz J, Noguera-Julian M 2016 Whole-genome sequencing of two *Bartonella bacilliformis* strains. Genome Announc 4:e00659-16. doi:10.1128/genomeA.00659-16. 27389274 PMC4939791

[7] Tarazona D, Padilla C, Cáceres O, Montenegro JD, Bailón H, Ventura G, Mendoza G, Anaya E, Guio H 2013 Whole genome sequencing and comparative analysis of *Bartonella bacilliformis* strain INS, the causative agent of Carrion’s disease. Genome Announc 1:e00053-12. doi:10.1128/genomeA.00053-12. PMC3569277 23409255

[8] Slack JM, Mitchell PD, Baumann B 1966 Studies on the pathogenicity and related characteristics of *Bartonella bacilliformis*. Morgantown West Virginia University Medical Center, Morgantown, WV https://apps.dtic.mil/dtic/tr/fulltext/u2/484102.pdf.

[9] Brenner DJ, O’Connor SP, Hollis DG, Weaver RE, Steigerwalt AG 1991 Molecular characterization and proposal of a neotype strain for *Bartonella bacilliformis*. J Clin Microbiol 29:1299–1302. 1715879 10.1128/jcm.29.7.1299-1302.1991 PMC270104

[10] Riess T, Dietrich F, Schmidt KV, Kaiser PO, Schwarz H, Schäfer A, Kempf VAJ 2008 Analysis of a novel insect cell culture medium-based growth medium for *Bartonella* species. Appl Environ Microbiol 74:5224–5227. doi:10.1128/AEM.00621-08. 18567689 PMC2519262

[11] Andrews S, Lindenbaum P, Howard B, Ewels P 2010 FastQC: a quality control tool for high throughput sequence data. http://www.bioinformatics.babraham.ac.uk/projects/fastqc.

[12] Wick RR, Judd LM, Gorrie CL, Holt KE 2017 Unicycler: resolving bacterial genome assemblies from short and long sequencing reads. PLoS Comput Biol 13:e1005595. doi:10.1371/journal.pcbi.1005595. 28594827 PMC5481147

[13] Li H 2018 Minimap2: pairwise alignment for nucleotide sequences. Bioinformatics 34:3094–3100. doi:10.1093/bioinformatics/bty191. 29750242 PMC6137996

[14] Tatusova T, DiCuccio M, Badretdin A, Chetvernin V, Nawrocki EP, Zaslavsky L, Lomsadze A, Pruitt KD, Borodovsky M, Ostell J 2016 NCBI Prokaryotic Genome Annotation Pipeline. Nucleic Acids Res 44:6614–6624. doi:10.1093/nar/gkw569. 27342282 PMC5001611

